# Potential Seeding From Fine-Needle Aspiration of an Axial Osteosarcoma: A Case Report

**DOI:** 10.3389/fvets.2022.847933

**Published:** 2022-04-29

**Authors:** Tasha Faletti, Bernard Seguin, Laura Elizabeth Selmic, Janis Lapsley, Deanna Worley, Maureen Griffin, Giovanni Tremolada

**Affiliations:** ^1^Department of Veterinary Clinical Sciences, The Ohio State University, Columbus, OH, United States; ^2^College of Veterinary Medicine Flint Animal Cancer Center, Colorado State University, Fort Collins, CO, United States

**Keywords:** seeding, canine (dog), osteosarcoma, fine needle aspiration (FNA), oncology

## Abstract

This report describes the first potential case of seeding after fine-needle aspiration (FNA) of a rib osteosarcoma in a dog. An 8-year-old, 28-kg female spayed Golden Retriever was presented to her primary veterinarian with a 3-week history of a 3-cm firm, unpainful, immobile mass arising from the 9th rib. The mass was aspirated and submitted for cytological examination. A subcutaneous nodule developed several days after the FNA was performed in a location immediately overlying but distinct from the primary rib tumor on palpation. Both the primary mass and the newly diagnosed subcutaneous nodule were biopsied and were consistent with an osteosarcoma. Although it cannot be ruled out that the subcutaneous lesion was metastatic, seeding was a reasonable explanation based on where the new mass was located and how quickly it appeared after the FNA was performed. The aim of this case report was to describe the possibility of tumor seeding during FNA for osteosarcoma. It is the authors' opinion that utility of cytological diagnosis of bone tumors outweighs the risk of possible seeding and should continue to be used as a routine diagnostic test for the diagnosis of aggressive bone lesions.

## Introduction

Osteosarcoma (OSA) is an aggressive tumor accounting for 85% of primary bone tumors in dogs (1). This type of tumor usually affects large to giant breed dogs at a median age of 7 years (2). While 75 percent of OSA occurs in the appendicular skeleton, cases of OSA originating from the axial skeleton have been reported (3–6). OSA most commonly metastasizes to the lungs and other bones; however, visceral, subcutaneous, and cutaneous metastases have also been reported (7–10).

A preoperative diagnosis of bone tumors can be obtained by performing fine-needle aspiration (FNA) or incisional biopsy of the lesion. Collecting an FNA sample *via* ultrasound guidance has been described and may increase the ability of obtaining a diagnostic sample (11). While needle track seeding from carcinomas has been reported in dogs, cats, and humans (12–19), to the authors' knowledge, seeding from an FNA of OSA has not previously been described before in the human or veterinary literature.

## Case Presentation

An 8-year-old, 28-kg female spayed Golden Retriever was presented to her primary veterinarian with a 3-week history of a 3-cm firm, unpainful, immobile, and ill-defined mass on the left lateral thorax. No other abnormalities were noted on physical examination. Radiographs of the thorax were obtained to identify the origin of the mass and revealed an expansile, lytic, and proliferative bone lesion arising from the 9th rib. A blind FNA sample was collected for cytology and evaluated by a veterinary clinical pathologist. The pathological description was consistent with a malignant neoplasia with primary differentials including osteosarcoma, chondrosarcoma, and plasma cell tumor. Three-view thoracic radiographs revealed no evidence of pulmonary metastatic disease. The dog was then referred to a specialty hospital for further diagnostics and discussion of treatment options.

Three weeks following the original presentation, the dog was assessed in our institution. On physical examination, a new 1-cm erythematous, freely movable, and subcutaneous nodule located directly over the rib mass was noted. According to the owner, the nodule developed a few days after the FNA sample of the mass was obtained. The size of the rib mass had also increased from 3 to 5 cm. The remainder of the physical examination was otherwise unremarkable. Bloodwork was performed with biochemical analysis showing a mild elevation in alkaline phosphatase of 153 IU/L (15–140 IU/L), with no other abnormalities, and complete blood cell count was within normal limits. Cytology of both the subcutaneous nodule and the rib mass was performed *via* blind FNA and showed the presence of a malignant neoplasia with certain cellular features suspicious for osteoblasts in both samples, but other tumor types such as histiocytic sarcoma and hemangiosarcoma could not be ruled out. A biopsy for histopathology was recommended.

Thoracic radiographs were offered to the client for staging purposes but were declined at this time because of a preference to have them performed by the primary veterinarian. Flouride-18 fludeoxy glucose positron emission tomography and computed tomography (18F-FDG PET/CT) (Phillips Gemini TF Big Bore 16-slice; Phillips North America, Cambridge, MA, United States) was also offered to more thoroughly stage the patient but was declined for financial reasons. Three-view thoracic radiographs were obtained the following day by the primary veterinarian. The images did not show evidence of pulmonary metastatic disease, intrathoracic lymphadenopathy, or mediastinal masses.

**Figure 1:**
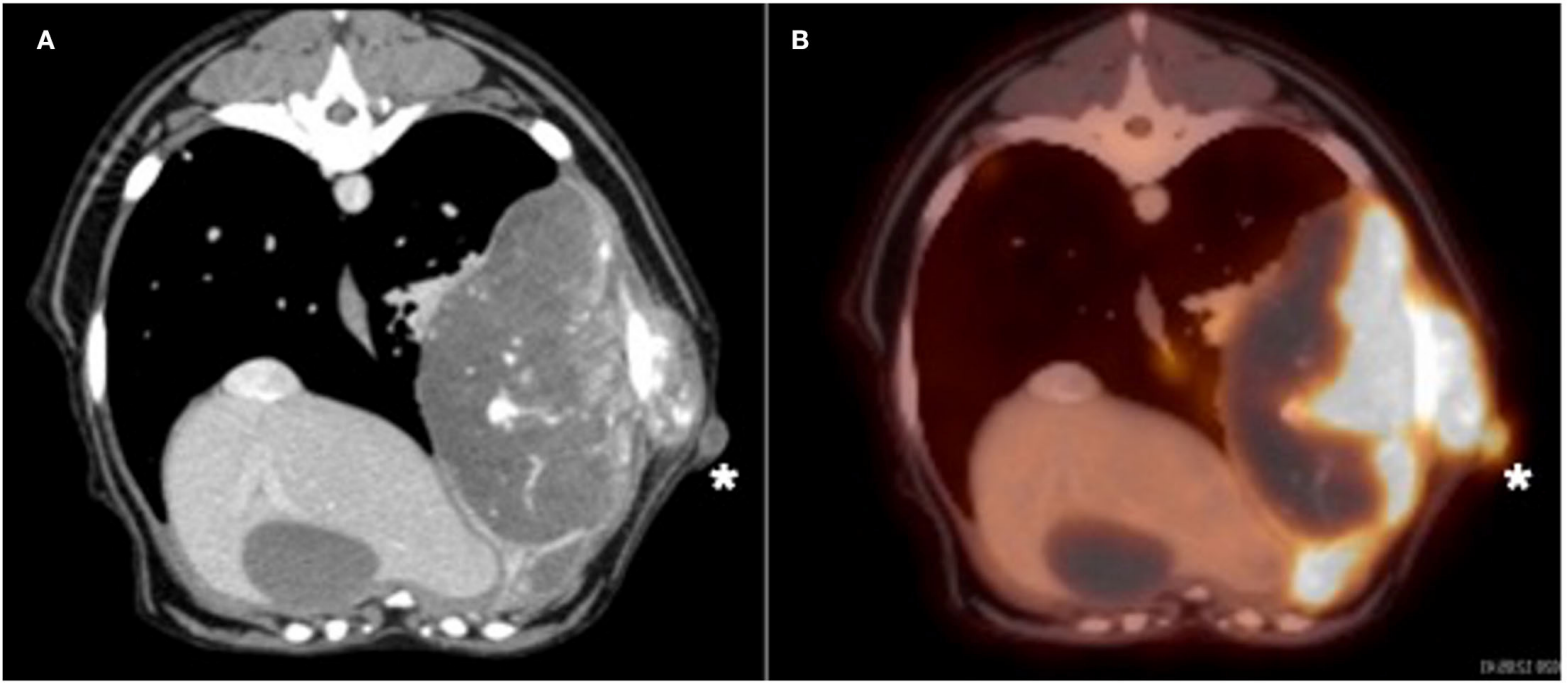
**(A)** Transverse CT and positron emission tomography (PET)/CT **(B)** images of the large left 9th rib lesion and the subcutaneous nodule (white asterisk). Note the marked avidity of bone associated with the primary bone lesion and the subcutaneous nodule after intravenous administration of flouride-18 fludeoxy glucose (18F-FDG) in the PET/CT image.

**Figure 2:**
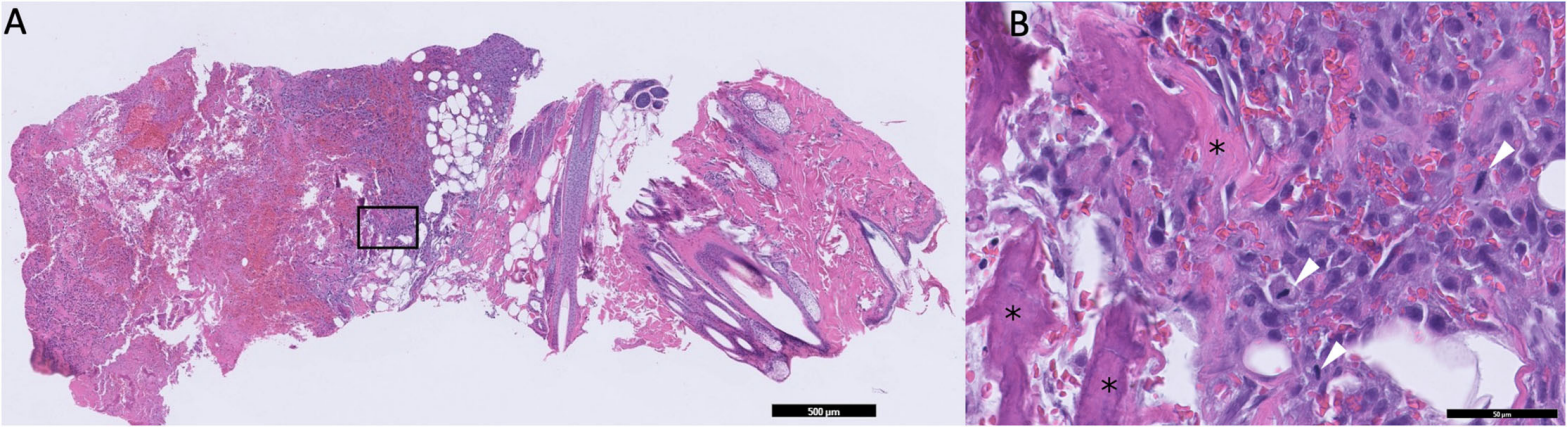
**(A)** Low magnification microphotograph of the suspected seeded lesion in the subcutaneous tissue. The black box represents the area of increased magnification in B. **(B)** High-magnification microphotograph of the neoplastic tissue. Areas containing osteoids are marked with asterisks. Tumor cells undergoing mitosis are marked with white arrowheads.

Due to the absence of lung metastasis, the owner decided to return to our institution 2 weeks later to have an 18F-FDG PET/CT performed. On physical examination, the mass appeared to be progressive in size (6.5 cm). The dog also had a new onset of exercise intolerance. A total body F-18FDG PET/CT was performed after injection of 5 microcuries of 18F-FDG. The study showed a large, aggressive, osteolytic, and osteoproductive mass, with high avidity [degree of uptake of 18F-FDG measured as maximum standardized uptake value (SUV_max_) 16.3] centered around the left 9th rib. An avid subcutaneous mass separated from the primary rib lesion was identified (Figure 1). Additionally, an indistinct soft tissue nodule with a mineral center in the caudal mediastinum (SUV_max_ 7.4), a very avid left sternal lymph node (SUV_max_ 9.8) compared to the right (SUV_max_ 1.9), and many small soft tissue pulmonary nodules throughout the pulmonary parenchyma were identified. The remainder of the study found no significant abnormalities. During the same anesthetic event, Tru-cut biopsies with a 16-gauge needle were obtained from the mass on the 9th rib, as well as the associated subcutaneous nodule. No aspirations of the other abnormalities noted on imaging were performed. The histopathology of the subcutaneous nodule showed haired skin and subcutaneous adipose tissue with an inner neoplastic osseous core (Figure 2). Cells in the neoplastic region were stellate to spindle with eosinophilic cytoplasm. Mitotic index was moderate with 1–4 mitotic figures per high-power field. These findings were consistent with extra-skeletal osteosarcoma. The histopathology of the rib mass had similar findings, and the mass was classified as osteosarcoma.

Due to the advanced stage of the tumor, palliative radiation therapy with or without the addition of chemotherapy was offered to the client but was declined. The patient was maintained on NSAIDs for pain control. Two weeks following the last appointment, the patient was humanely euthanized due to rapid progression of clinical signs causing decreased quality of life combined with a grave prognosis.

## Discussion

Cytology is a practical and useful technique for obtaining a definitive diagnosis of many tumor types in small animals (20). Bone tumors can be accurately diagnosed using cytological evaluation, and the technique has been shown to be useful in differentiating between malignant and nonmalignant lesions (21, 22). The diagnostic accuracy of cytology compared to histology for bone tumors has been reported to be 83–92% in two studies (23, 24). Compared to incisional bone biopsy, fine needle aspiration with cytology is associated with lower risk of pathologic fracture, does not require special instrumentation, and results in faster diagnosis. To increase the chance of obtaining a diagnostic sample, FNA can be performed under ultrasound guidance (11).

The subcutaneous lesion identified in this patient cannot be ruled out as metastasis from the primary rib lesion. However, seeding was thought to be a reasonable explanation, since the lesion appeared a few days after the FNA was performed and was in a location immediately overlying but distinct from the primary rib tumor. This theory is supported by the findings from a case report where seeding was detected 2 weeks after obtaining an FNA sample in a cat with a lung carcinoma (25). Needle tract seeding is a reported complication of FNA of carcinomas in small animals and in humans (12–19, 25), but it has not been reported for sarcomas. The size of the needle used to obtain samples may play a role in the risk of seeding. Needles larger than 23-gauge are reported to increase the risk of seeding in human patients with thyroid carcinoma (26). Unfortunately, the size of the needle used in our case was not reported in the medical record for this case.

The possibility of seeding after core biopsies using Tru-cut needles in humans with sarcomas has been briefly described in case reports and small case series, but the low sample size and limited patient follow-up do not allow for definitive conclusions on the true frequency of this event (27, 28). One human study analyzed the contamination of biopsy tracts from primary malignant bone tumors. The results of this study showed 11.4% contamination and correlated with local recurrence of neoplasia (29). Another human study published in 2015 showed that 20% of patients had tumor foci around the biopsy tract line when needle core biopsy was performed for diagnosis (30). Based on these findings, it is important for clinicians to perform incisional biopsies in a location that will allow for excision of the biopsy tract during tumor removal.

The aim of this case report was to describe, for the first time in veterinary medicine, the possibility of tumor seeding during FNA for OSA. It is the authors' opinion that utility of cytological diagnosis of bone tumors outweighs the risk of possible seeding and should continue to be used as a routine diagnostic test for the diagnosis of aggressive bone lesions.

## Data Availability Statement

The original contributions presented in the study are included in the article/supplementary material, further inquiries can be directed to the corresponding author/s.

## Ethics Statement

Ethical review and approval was not required for the animal study because no experimental procedures were performed. Written informed consent for participation was not obtained from the owners because no experimental procedures were performed. The owner consented to all diagnostics performed.

## Author Contributions

TF wrote the first draft. GT, BS, DW, and MG managed the patient and contributed to conception of the case report. All authors contributed to manuscript revision, read, and approved the submitted version.

## Conflict of Interest

The authors declare that the research was conducted in the absence of any commercial or financial relationships that could be construed as a potential conflict of interest.

## Publisher's Note

All claims expressed in this article are solely those of the authors and do not necessarily represent those of their affiliated organizations, or those of the publisher, the editors and the reviewers. Any product that may be evaluated in this article, or claim that may be made by its manufacturer, is not guaranteed or endorsed by the publisher.
